# Feedback Timing Modulates Probabilistic Learning in Adults with ADHD

**DOI:** 10.1038/s41598-018-33551-3

**Published:** 2018-10-19

**Authors:** Yafit Gabay, Elham Shahbari-Khateb, Avi Mendelsohn

**Affiliations:** 1 Department of Special Education, University of Haifa, Haifa, Israel; 2 Edmond J. Safra Brain Research Center for the Study of Learning Disabilities, University of Haifa, Haifa, Israel; 3 Sagol Department of Neurobiology, University of Haifa, Haifa, Israel; 4 Institute for Information Processing and Decision Making, University of Haifa, Haifa, Israel

## Abstract

Attention deficit hyperactivity disorder (ADHD) has been associated primarily with executive function deficits. Emerging findings suggest, however, that procedural learning may be compromised as well. To this effect, we recently showed that feedback-based procedural learning is selectively impaired in ADHD, results that coincide with dopaminergic alterations associated with ADHD. Key questions, however, remain unresolved, among which are the learning conditions that may improve procedural learning in ADHD. Here we examined feedback-based probabilistic learning during conditions that engage procedural and declarative learning systems to different degrees, depending on feedback timing. ADHD and control participants carried out a probabilistic learning task in which they were required to learn to associate between cues and outcomes, where outcomes were presented either immediately or with a short/long delays. Whereas performance in probabilistic learning in ADHD participants was comparable to controls in delayed feedback conditions, during both learning and test phases, their performance diminished when feedback was immediate. Furthermore, ADHD symptom severity was negatively correlated with the ability to learn from immediate feedback. These results suggest that feedback-based probabilistic learning can be improved in ADHD, provided appropriate conditions. By shifting the load from midbrain/striatal systems to declarative memory mechanisms, behavioral performance in ADHD populations can be remediated.

## Introduction

Attention deficit hyperactivity disorder (ADHD) is one of the most common neurodevelopmental disorders, with an estimated prevalence ranging between 3–5% of the general population. It is characterized by age-inappropriate levels of sustained attention and impulse control and heightened restlessness that are present across multiple environments^1^. The disorder has significant educational, emotional, as well as social developmental deficits to those affected. ADHD has been mostly associated with impairments in executive functions^2^. Yet, burgeoning literature indicates that motivational and reward-related processes may be affected as well^3–7^. Children and adolescents with ADHD display an enhanced sensitivity to rewards compared to their non-ADHD counterparts^8,9^, and tend to prefer small immediate rewards over larger delayed ones^10–13^. Reward-based learning is central to the formation of certain non-declarative or procedural memories, including skill acquisition and habit formation. Ongoing research suggests that procedural learning mechanisms in those with ADHD are impaired^14^, and particularly when involving learning from feedback^15,16^.

Consistent with the latter observations, the procedural learning deficit hypothesis (PLD) postulates that a selective dysfunction in the procedural learning system is associated with a family of neurodevelopmental disorders, including ADHD^17–20^. This view highlights the involvement of procedural learning systems, through which knowledge is acquired across multiple repetitions, is expressed through performance rather than recollection, and is rigid and difficult to verbalize. This form of learning, as opposed to declarative knowledge formation, may gradually lead to the formation of skills (the process of improving behaviors through repeated practice) and of habits, based on trial-and-error learning of stimulus-response associations^21^. Whereas the formation of declarative memories relies heavily on the integrity of medial temporal lobe structures, gradual procedural learning requires the engagement of the basal ganglia, and particularly the striatum^21^. According to the PLD framework, the striatal-based procedural learning system is hypothesized to be impaired in ADHD, whereas hippocampal-based declarative learning is assumed to remain intact^17–19^, and in some cases enhanced, possibly serving a compensatory role^20^.

Consistent with this hypothesis, anatomical and functional alterations of basal ganglia structures and the hippocampus were documented in ADHD populations^14^. For example, the overall volume of each of the basal ganglia nuclei (caudate, putamen, and globus pallidus) are reduced among individuals with ADHD compared with neurotypicals, and particularly in the putamen^22,23^. Along with the indications of decreased basal ganglia volume in ADHD, reduced activation in these nuclei are also observed among ADHD individuals across a variety of tasks and ages^6,24^. Neurochemically, ADHD individuals were found to possess lower striatal dopamine levels, and both children and adults with ADHD were shown to have an abnormally high density of dopamine transporters (DAT’s)^25,26^. Pertinent to the PLD framework, there is evidence to suggest neural compensation in brain structures involved in declarative learning and memory^27^. For example, larger hippocampus volumes bilaterally, especially in more anterior portions of the hippocampus, were observed in children and adolescents with ADHD^27^. Additionally, symptom severity in ADHD was shown to be negatively linked with these portions of the hippocampus, findings that were attributed to a hippocampal-based compensatory role^27,28^.

At the behavioral level, there is evidence that procedural learning is disrupted in ADHD. For example, studies using the serial reaction time task revealed fewer oculomotor anticipations and a more inconsistent progress of learning in children with ADHD^29,30^. Furthermore, impairments in motor skill learning in ADHD are observed not only during the acquisition phase but also during offline processes necessary for skill consolidation^31–34^. Procedural learning problems in ADHD were also found to be extended beyond the motor domain and include also the ability to learn complex artificial language rules^35^. Similarly, habitual learning, as studied by tasks that model habit formation in humans, such as visual category learning^15^ and incremental learning of stimulus-response associations guided by corrective feedback^16^, are reduced in ADHD. Lastly, procedural reinforcement learning (i.e. the ability to learn and modify behaviors based on their association with positive and negative outcomes) is also affected in those with ADHD. In particular, studies have shown that ‘go learning’ (i.e., the ability to learn from positive feedback) is disrupted in ADHD^36^.

Overall, accumulating evidence points to substantial alterations of particular learning systems in individuals with ADHD, consistent with the PLD hypothesis^17–20^. However, it is valuable to characterize not only the instances wherein learning processes fail in ADHD, but also to highlight the conditions that may improve learning. Since there is reason to believe that relatively intact functions/circuits can play important compensatory roles in neurodevelopmental disorders, capitalizing on these dissociations may be of significant value^20^.

## Probabilistic Learning in ADHD

Manipulating task features can significantly influence the neural systems engaged and as a result the behavioral learning outcomes^37^. One example for this is the weather prediction task, WPT^38^, whereby participants are required to predict an outcome (the weather) based on different combinations of geometric features presented on cards based on corrective feedback. It has been shown that although in early stages of learning activity is observed in MTL, as learning progresses there is a shift towards neural systems involved in procedural learning (basal ganglia)^39^. In a slightly different version of the task, participants are encouraged to learn the associations between simultaneously presented cues and outcomes by mere observation. Neuroimaging studies have shown that under this manipulation, the striatum is much less engaged than in the typical task version^40^. Consistently, patients whose condition affects dopaminergic input to the striatum (e.g., Parkinson’s disease) exhibit impaired learning when trained under feedback-dependent conditions^41^, while maintaining intact performance via observational training^42^. A similar observation was found in patients with Huntington’s disease, characterized by a loss of cells in the striatum^43^. Using positron emission tomography (PET), Wilkinson, *et al*.^44^ observed dopamine release in the right ventral striatum of healthy participants when performing the WPT based on trial-by-trial feedback, but not in an observational task devoid of feedback. These studies provide evidence that behavioral performance and neural engagement are sensitive to task demands, and that patients with dopamine-related malfunctions can benefit from specific task conditions. Here we assessed whether ADHD individuals may similarly benefit from particular task conditions in probabilistic learning by manipulating feedback timing.

Converging evidence from animal models and human studies suggests that the timing of feedback determines the engagement of hippocampal vs. striatal-based learning. In particular, the ability to learn from immediate feedback seems to rely heavily on fast phasic dopamine releases in the striatum in response to feedback^45^. These responses are considered to promote learning by facilitating cortico-striatal plasticity, presumably by reinforcing reward-related associations to relevant responses or stimuli^46^. Introducing a temporal gap between responses and rewards, however, can fundamentally change striatal responses and strengthen inappropriate synapses, indicating this mechanism is not suitable for learning under delayed feedback conditions^47,48^. Using model-based functional imaging (fMRI), Foerde and Shohamy^49^ exhibited direct support for the role of the hippocampus in delay feedback conditions. They demonstrated that immediate feedback trials in a probabilistic learning task resulted in striatal engagement in healthy individuals, as opposed to hippocampal activation when feedback was delayed by several seconds. Additionally, learning in this task was impaired in patients suffering from neural diseases affecting the dopaminergic functioning in the striatum, but was rescued if feedback was delayed by a few seconds^50^. That different task features can determine the involvement of particular learning systems is imperative to the domain of neurodevelopmental disorders and may be particularly relevant to the understanding and remediation of ADHD, which is accompanied by specific learning impairments. Consistent with the above-mentioned findings, we have recently shown that probabilistic learning is selectively disrupted in individuals with ADHD^16^. Specifically, we demonstrated that compared to controls, high-functioning young-adult university students diagnosed with ADHD were selectively impaired in learning the WPT from feedback, whereas learning by observation was intact.

As mentioned above, recent observations suggest that probabilistic learning of stimulus-response associations among populations with basal ganglia dysfunctions can be remediated by manipulating certain training conditions^50^. These findings call into question whether probabilistic learning impairments in ADHD could be remediated based on the timing of task features during the training experience. Given that ADHD is characterized by differential functioning of learning systems, we hypothesized that delaying feedback will benefit feedback-based learning of participants with ADHD.

## Results

Following prior studies e.g.^38,40,42^, participants who failed to perform above chance level at test phase in each one of the feedback conditions were excluded from further analysis. Based on this criterion, four controls and one ADHD participant were excluded from further analysis. We first compared accuracy and RT performance of ADHD and control participants during the learning and test phases and tested correlations between performance and ADHD measures.

### Accuracy analyses

#### Feedback-based learning across training-trial blocks

**Figure 1:**
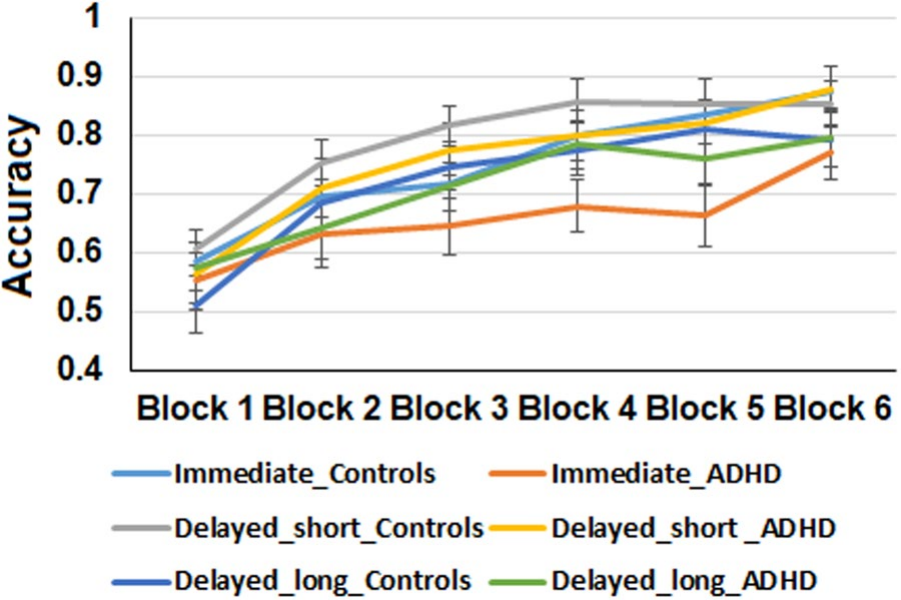
Accuracy performance of the two groups during the learning phase across all feedback conditions. Error bars represent standard error.

An analysis of variance (ANOVA) was conducted with block (1–6) and feedback type (immediate, delayed-short, delayed-long) as within-subject factors, and group (ADHD vs. Control) as a between-subjects factor using mean proportion of correct responses during the learning phase as the dependent variable. Results are presented in Fig. 1. The main effect of the group was not significant, (*F* (1, 47) = 2.07, *p* = 0.15, *η*_*p*_² = 0.04). There was a significant main effect of block, (*F* (5, 235) = 44.52, *p* < 0.01, *η*_*p*_² = 0.48), indicating that participants improved at predicting the associated letters leading to correct outcomes across trials. Further analysis revealed a significant linear trend (using a linear contrast test – (*F* (1, 47) = 111.80, *p* < 0.01), such that accuracy parametrically improved with training. The main effect of feedback was also significant (*F* (2, 94) = 4.35, p < 0.01, *η*_*p*_² = 0.08), indicating that overall, participants performed better during delayed-long feedback trials compared with all other feedback trials (*F* (1, 47) = 11.26, *p* < 0.01, *η*_*p*_² = 0.48), yet no differences were observed between delayed-short and immediate feedback trials, *F* < 1. However, when testing each group separately, a main effect for feedback across blocks was not significant for controls (*F* (2, 50) = 2.33, *p* = 0.11), but was significant for ADHD, (*F* (2, 52) = 5.95, *p* < 0.01), indicating that only in the ADHD group was there a difference between the feedback conditions. As such, the superior performance was apparent during the long delayed feedback condition specifically in the ADHD group. Although the group by block interaction was not significant (*F*(5, 235) = 1.26, p = 0.28; *η*_*p*_² = 0.02), the feedback type by group interaction was significant (*F* (2, 94) = 3.27, p < 0.05, *η*_*p*_² = 0.06), such that ADHD participants performed significantly more poorly than controls during the immediate feedback trials, (*F* (1, 47) = 6.58, *p* < 0.01), while no difference was observed during the delayed feedback trials, (*F*’s < 1 for both delayed-long and delayed-short feedback trials). The interaction of feedback, group and block was not significant, *F* < 1. Out of theoretical interest, the performance accuracy in the training blocks was also examined separately as a function of group for each feedback type separately. For the immediate feedback condition, the interaction of group by block was marginally significant, (*F* (1, 23) = 1.93, *p* = 0.09). Further analysis suggests that the linear trend differed significantly between the groups (*F* (1, 47) = 6.32, *p* < 0.05) with ADHD participants presenting less improvement across blocks. For the other training conditions, the block by group interaction was not significant (all *F* < 1).

**Figure 2:**
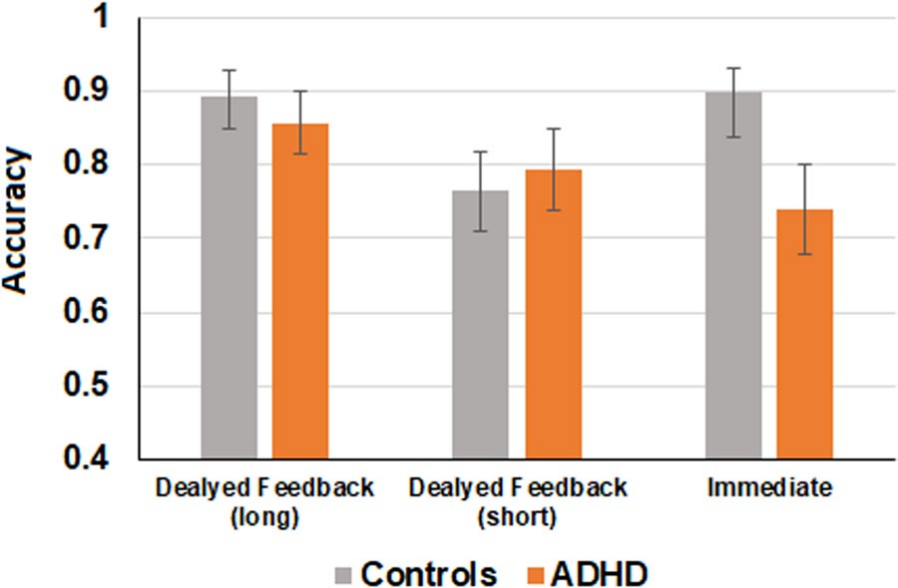
Accuracy performance of the two groups during the test phase across all feedback conditions. Error bars represent standard error.

#### Test phase

An analysis of variance (ANOVA) test was conducted using feedback type (immediate, delayed-short, delayed-long) as a within-subjects factor and group (ADHD vs. Control) as a between-subjects factor, and mean proportion of correct responses during the test phase as the dependent variable. Results are presented in Fig. 2. The main effect of group was not significant, (*F* (1, 47) = 1.1, *p* = 0.29, *η*_*p*_² = 0.02) whereas the main effect of feedback type was significant, (*F* (2, 94) = 3.57, *p* < 0.05, *η*_*p*_² = 0.07). Importantly, the group by feedback type interaction was significant, (*F* (2, 94) = 3.54, *p* < 0.05, *η*_*p*_² = 0.07), stemming from the fact that participants with ADHD performed significantly poorer than control participants in the immediate feedback trials (*F* (1, 47) = 5.44, *p* < 0.05), whereas no significant differences were observed for the delayed feedback trials (all *F’s* < 1 for the delayed-long and delayed short feedback trials).

**Figure 3:**
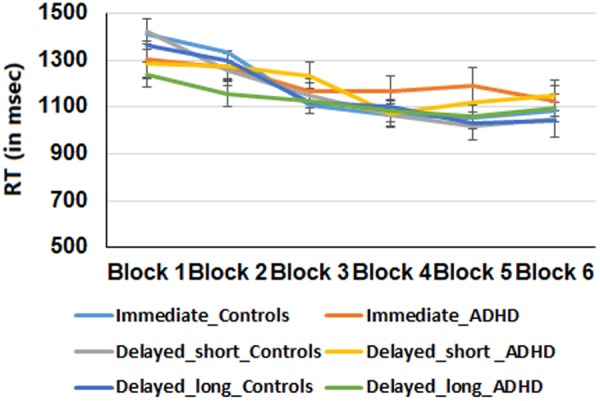
Accuracy performance of the two groups during the learning phase across all feedback conditions. Error bars represent standard error.

### RT analyses

#### Feedback-based learning across training-trial blocks

We further examined whether RT effects were apparent during the course of learning. To this end, a mixed-effects ANOVA was conducted, using block (1–6) and feedback type (immediate, delayed-short, delayed-long) as within-subject factors, and group (ADHD vs. Control) as a between-subjects factor using mean reaction-times during the learning phase as the dependent variable. Results are presented in Fig. 3. There was a significant main effect of block, (*F* (5, 235) = 17.91, *p* < 0.01, *η*_*p*_² = 0.27), but not for group, indicating that participants from both groups improved in response latencies for predicting the associated letters leading to outcomes across trials. Further linear contrast tests revealed a significant linear trend, such that as training progressed, RTs diminished (*F* (1, 47) = 28.13, *p* < 0.01). The main effect of feedback was also significant (*F* (2, 94) = 3.43, p < 0.05, *η*_*p*_² = 0.06), indicating that overall, participants responded faster during delayed-long feedback trials compared with the other two feedback conditions (*F* (1, 47) = 6.06, *p* < 0.05), yet no differences were observed between delayed-short and immediate feedback trials. Although the group-by-feedback type interaction was not significant, (*F* (2, 94) = 1.86, p = 0.16, *η*_*p*_² = 0.03), the group by block interaction was significant (*F* (5, 235) = 2.7, p < 0.05; *η*_*p*_² = 0.05), such that RT improved in a linear manner as training progressed for both groups, but was more pronounced in the control group (*F* (1, 47) = 26.13, *p* < 0.01) compared with the ADHD group (*F* (1, 47) = 5.57, *p* < 0.05). The interaction of feedback type, group and block and the interaction of block by feedback were not significant.

**Figure 4:**
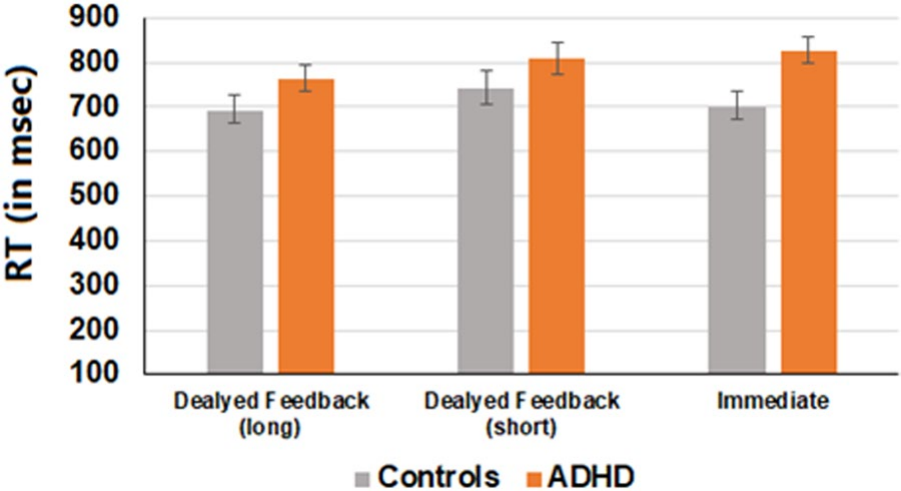
RT performance of the two groups during the test phase across all feedback conditions. Error bars represent standard error.

#### Test phase

To examine possible differences in reaction time between the groups and feedback conditions, an ANOVA test was conducted using feedback type (immediate, delayed-short, delayed-long) as a within-subjects factor and group (ADHD vs. Control) as a between-subjects factor, and mean reaction time during the test phase as the dependent variable. Results are presented in Fig. 4. A main effect for group was marginally significant, (*F*(1, 47) = 3.99, *p* = 0.051, *η*_*p*_² = 0.07), such that the ADHD group was somewhat slower than the control group regardless of feedback timing. A main effect for feedback type was found (*F* (2, 94) = 3.57, *p* < 0.05, *η*_*p*_² = 0.12), indicating that participants were relatively faster in the delayed feedback condition compared with the other feedback conditions, (*F* (1, 47) = 6.06, *p* < 0.05). The group by feedback type interaction was marginally significant, (*F* (2, 94) = 3.04, *p* = 0.052, *η*_*p*_² = 0.06), stemming from the fact that participants with ADHD were significantly slower than control participants in the immediate feedback trials *F* (1, 47) = 8.76, *p* < 0.01, and comparable to the delayed feedback trials (*F*(1, 47) = 1.6, *p* = 0.206 for the delayed vs. long feedback and (*F* (1, 47) = 2.58, *p* = 0.11 for delayed vs. short feedback trials).

**Figure 5:**
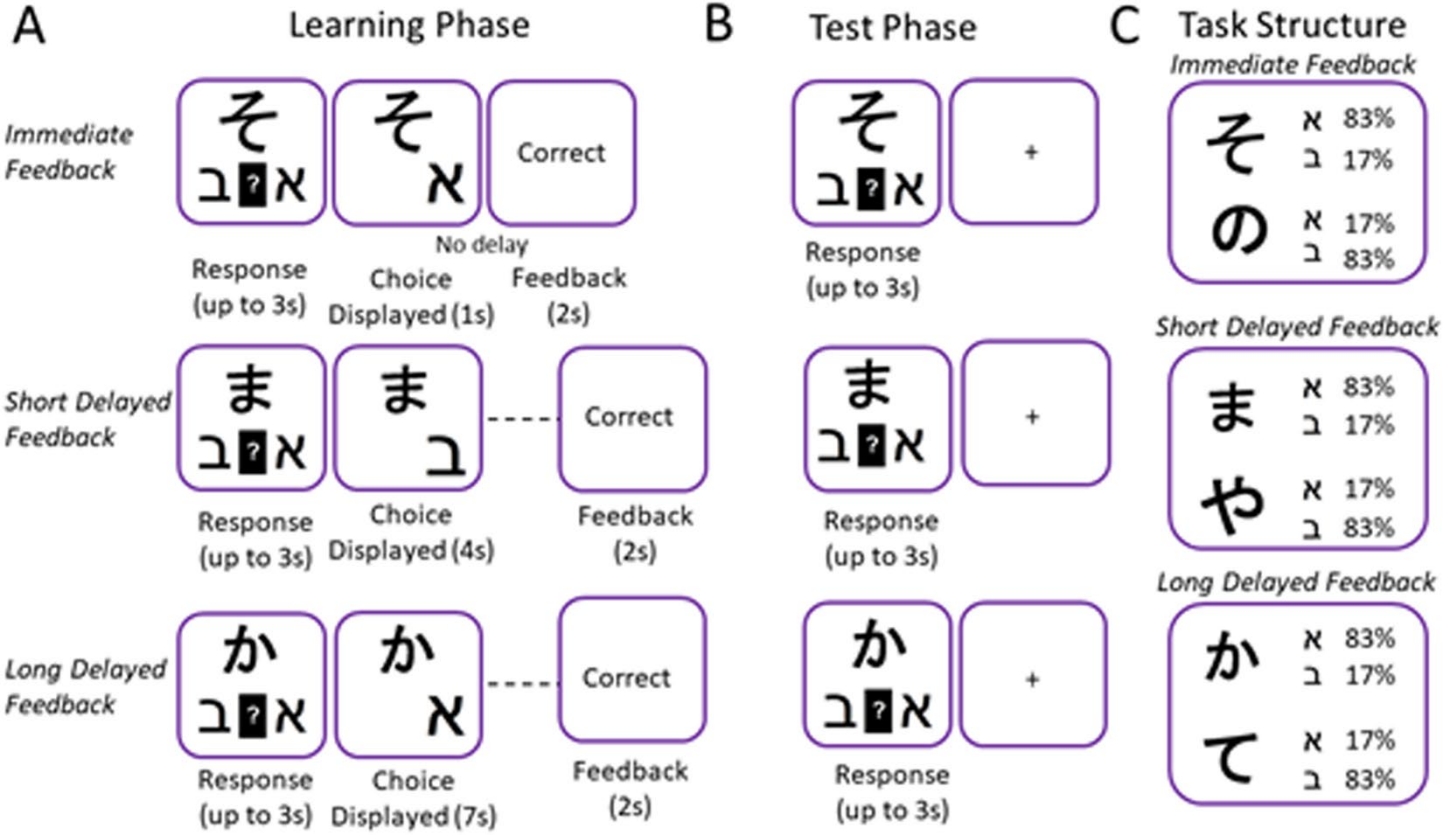
Probabilistic leaning task adapted from Foerde and Shoamy (2011). Participants used trial-by-trial feedback to learn which Hebrew letter six different Asian characters were associated with (Learning phase, **A**). For one set of Asian characters’ feedback was presented immediately (0 s) after choice displayed. For another set of Asian characters’ feedback was presented with a short (3 s) or long delay (6 s) after choice displayed. After the learning phase was ended, participants completed a probe task in which they continued to make predictions about associations between letter and characters (Test phase, **B**). However, they no longer received feedback and the timing of all trials events was equal across trial types. Each Asian character was associated with one Hebrew letter on 83% of trials and with the other Hebrew letter on 17% of trials (**C**).

#### Relationship between learning and ADHD symptoms

In addition to the group analyses reported above, we also examined the relationship between learning and test accuracy performance and ADHD symptoms, as measured by the DSM questionnaire, separately for each one of the different training conditions.

#### Correlations between ADHD symptoms and test performance

A significant negative correlation (*r* = −0.38) was found between inattentive symptoms and accuracy test performance of immediate feedback trials. There were no significant correlations for the other feedback training conditions.

#### Correlations between ADHD symptoms and learning index

A difference score was calculated by subtracting the mean of the last block from the mean of the first block during the learning phase. The learning score for the immediate feedback condition showed a significant negative correlation with inattentive and hyperactive symptoms (*r* = −0.38 and *r* = −0.39, respectively). There were no significant correlations for the other feedback training conditions.

## Discussion

Recent observations suggest that probabilistic learning of stimulus-response associations among populations with dopaminergic and striatal dysfunctions could be remediated by manipulating specific aspects of the training procedure^51,52^. This hypothesis was put here to test by examining whether probabilistic learning impairments in ADHD individuals would similarly depend on the duration of feedback delay during the training experience. Given that procedural learning systems, but not necessarily other learning systems, are disrupted in ADHD, we expected that by providing conditions that may engage extra-basal ganglia systems, probabilistic learning impairments of ADHD patients would be alleviated. Indeed, we demonstrate that the ability to utilize immediate feedback in the context of probabilistic learning is disrupted in ADHD. Furthermore, we show that ADHD symptom severity is negatively correlated with the ability to learn from immediate feedback. Under delayed feedback conditions, however, we found that ADHD participants perform similarly to that observed in typical individuals.

Evidence of accuracy measures during the learning phase indicates that both groups of participants improved in learning as training progressed. However, ADHD participants demonstrated a specific deficit in immediate-feedback learning trials compared with neurotypical participants. One could argue that the diminished performance in the immediate condition might have stemmed from a reduced ability to process immediate feedback, rather than learn from it, in ADHD. However, no group differences were observed during the first block of immediate feedback trials of the learning phase, rendering this possibility less likely. Moreover, while a significant linear trend was observed in both groups as immediate feedback trials progressed, the improvement of the ADHD group was less pronounced. This state of affairs suggests that whereas control participants were able to utilize immediate feedback effectively in favor of learning, the same signal was less useful for ADHD individuals.

Performance differences in immediate feedback trials were most pronounced during the fifth and sixth blocks of the learning phase (although marginally significant differences were found in the third and fourth blocks as well). That ADHD performance deteriorated over time may indicate that increased fatigue or loss of motivation might have played a role in ADHD performance across time. However, fatigue, motivation or attention are expected to influence learning in ADHD regardless of feedback condition, which was not the case here. That group differences were most pronounced during the last blocks actually strengthens the notion of impaired ability to learn specifically from immediate feedback in ADHD, since learning is expected to be mostly manifested at later rather than initial blocks.

Findings from the test phase corroborate the performance during the learning phase. In the test phase, participants with ADHD were significantly impaired compared with neurotypicals on test trials that were previously assigned with immediate feedback during training, but not when feedback was delayed by a few seconds. This was manifested in both RT and accuracy measures. That ADHD participants were both slower and less accurate than the control group during the immediate feedback trials argue against speed-accuracy trade-offs. Notably, the abovementioned effects were found to correlate with psychometric measures, such that test/learning accuracy performance in immediate feedback trials was negatively related to ADHD symptom severity. Thus, the higher the ADHD symptom scores, the less the ability to learn from immediate feedback. Conversely, the ability to learn from delayed feedback trials was comparable across the two groups in both training and test phases. This observation suggests that feedback-timing modulation is sufficient to resolve the learning gap between the two groups.

Evidence from neuroimaging suggests the recruitment of the striatum and hippocampus in conditions of immediate vs. delayed feedback, respectively^49^. In support of this, patients with Parkinson’s disease are impaired in learning stimulus-response associations involving immediate, but not delayed feedback^51^. The current findings substantiate such a distinction, suggesting that striatal-based probabilistic procedural learning is impaired in ADHD, a finding that is consistent with the PLD framework^17–20^. It is nonetheless worthwhile to consider whether impaired performance during immediate feedback trials may have arisen from other sources of deficits attributed to ADHD rather than to a learning impairment. For example, temporal processing deficits have been observed in ADHD^53^, raising the possibility that forming associations between stimuli appearing in close temporal proximity may be harder to process in ADHD. This process is not likely to have influenced the current results, however, as temporal processing is typically examined using much shorter time intervals (i.e. milliseconds)^53^, and should be manifested already at the outset of learning and not only towards the end, as observed here. Considering the role that working memory – yet another process believed to be compromised in ADHD^54^ – may have played here, it should be noted that the chosen outcome and stimulus in the current task remained on screen during the delay period, thus minimizing working memory demands^49^.

The learning impairment from immediate feedback among ADHD participants supports theoretical claims that point to a link between procedural learning deficits and neurodevelopmental disorders. In particular, ADHD was suggested to arise from a selective impairment to the striatal-based procedural learning system^17,18,20,55^. Therefore, symptoms typically observed in those with ADHD (such as inattention, distractibility and lability in performance) could originate from difficulties in automatizing skills, resulting in a greater load on attentional resources. This perspective has gained support from behavioral studies demonstrating that procedural learning skills are affected in ADHD^15,16,29–31,56^, corroborated by neuroimaging data implicating structural and functional abnormalities in the basal ganglia and cerebellum in ADHD^14,57–60^. Consistent with this framework, we recently reported that feedback-based learning is selectively disrupted in ADHD, while the ability to learn by observation remains intact^16^. This type of learning, as the probabilistic learning task presented here, relies heavily on procedural learning systems^40^ that are mediated by dopaminergic innervation of the ventral striatum^44^. Here we emphasize that impairments in feedback-based probabilistic learning are not an all-or-none phenomenon, but are restricted to conditions presumed to rely on procedural learning systems. Devising task features that may affect learning among atypical populations (such as individuals with Parkinson’s disease) was found to be fruitful in previous investigations^50^, and the understanding of the behavioral strategies and neurobiological mechanisms of procedural learning can be illuminated by investigating special populations^61^. In the present study, we extend the notion that dopaminergic-based impairments such as Parkinson’s disease can benefit from changes of task features, to an ADHD population, for which the exact role that dopamine plays is less understood^25,26^. By enabling conditions that shift the load off midbrain/striatal systems, which may be compromised in ADHD, and enabling other, declarative memory systems to come into play, behavioral performance seems to become comparable between ADHD and control populations.

How do the present findings relate to neurobiological models of ADHD? There is evidence to suggest that reinforcement sensitivity in ADHD is altered^62^. For example, ADHD individuals tend to prefer immediate over delayed rewards, and tend to discount the value of future rewards more than non-ADHD individuals (i.e., show shorter delay-of-reinforcement gradients)^63–65^. Impaired reinforcement learning has also been observed among those with ADHD^36,66^, accompanied by abnormal neural processing of reinforcement information during learning^67–71^. Consistent with these findings, various models predict altered reinforcement learning in ADHD individuals^69^. Neurobiological models of ADHD, such as the Dynamic Developmental Theory (DDT)^72^ and the Dopamine Transfer Deficit (DTD)^73^ focus primarily on the midbrain dopamine system or on basal ganglia and associated routes (e.g., the Go/No-Go learning model). These models differ in their suggested accounts regarding the cause of altered reinforcement-learning behavior in ADHD. Whereas the DTD model argues that the phasic dopamine response to reinforcement is disrupted, the DDT model suggests that abnormally low tonic dopamine level may be responsible for the observed deficits. According to the Go/No-Go model of ADHD, reinforcement learning is hypothesized to be disrupted due to inadequate updating processes in working memory. This model assumes that a hypo-dopaminergic state is responsible for blunted dopamine responses to reward and impaired positive reinforcement learning. Notably, these proposed models predict impaired reinforcement learning in ADHD and according to both the DDT and DTD frameworks learning is expected to be better when reward is immediate rather than delayed^62^.

At first glance, it seems that our results conflict with the prediction of neurobiological models of ADHD as well as previous findings of reward processing in ADHD, in which immediate rewards are preferable than delayed ones. It should be noted however, that in contrast to temporal discounting tasks or delay aversion probes, the current probabilistic learning task does not offer the opportunity to decide between options of gaining immediate rewards vs. delayed ones. The current task involves a manipulation on the timing of feedback (either positive or negative), on which the participants have no control over. Thus, the relationship between probabilistic learning performance as a function of feedback timing and the value discounting of delayed outcomes is a not a straightforward one and should be directly examined in future studies. Although the present findings do not directly approve/refute neurobiological models of ADHD, they do support the PLD framework according to which learning that engages the striatum is presumed to be impaired but not learning that engages the hippocampus-based learning system.

The present results are consistent with previous findings suggesting that dopaminergic neurons are sensitive to the interval between stimuli and outcomes in the context of procedural learning. Thus, delaying the time intervals between stimuli and outcomes may still yield dopaminergic responses, but these will be irrelevant to the learning of the stimuli-outcome contingencies, and bare similarity to responses to unpredicted events^47^. The striatal system is thus less efficient in learning from delayed intervals, and learning in these cases seems to become more reliant on brain regions involved in declarative learning.

To conclude, whereas previous studies documented procedural learning deficits in ADHD, little attention has been paid to the conditions by which learning can be rescued. Based on the role of feedback timing in modulating memory systems, we examined the hypothesis that delaying feedback by a few seconds could resolve a learning gap observed in ADHD. Indeed, our findings suggest that while learning from immediate feedback in ADHD is impaired, it is intact when delaying feedback by a few seconds. The obtained results highlight the importance of task features in shaping learning behaviors among ADHD, and are consistent with the procedural learning account of neurodevelopmental disorders.

## Methods

### Participants

Twenty-six participants with attention deficit disorder and a matched control group of twenty-eight participants took part in the study for a total of 54 participants. All participants were native Hebrew speakers of Israeli Jewish ethnicity, enrolled as students of the University of Haifa, Israel, most of whom come from families of middle to high socioeconomic status. Diagnosis of a comorbid learning disability served as an exclusion criterion; a well-documented history of ADHD was the inclusion criterion for the ADHD group. Each individual received a formal diagnosis of ADHD performed by a pediatric neurologist and a positive screening for ADHD-based DSM-5 criteria. Namely, all participants in the ADHD group answered the DSM-5 criteria for ADHD, i.e., answering “YES” to at least 5 symptoms of the inattention criteria or the hyperactivity and impulsivity criteria (see Table 1 for group means). The control group was age-matched with the ADHD group, devoid of attention problems, and scored similarly to the ADHD in cognitive abilities, as evaluated using a series of cognitive tests that measured general intelligence, reading comprehension, and math skills. Written informed consent was obtained from all participants. The study was approved by the Institutional Review Board of the University of Haifa and was conducted in accordance with the Declaration of Helsinki; participants were remunerated for their participation.

**Table 1:** Psychometric Tests.

The following tests were administered, similar to the tests employed in the study of Gabay and Goldfarb (2017):	
1.	*Raven’s Standard Progressive Matrices test* (Raven^74^) – Non-verbal intelligence was assessed by the Raven’s-SPM test. This task requires participants to choose the item from the bottom of the figure that would complete the pattern at the top. The maximum raw score is 60. Test reliability coefficient is 0.9
2.	O*ne-minute Test for Words*^77^ – *R*eading skills were examined by the One Minute Test for Words which assesses the number of words accurately read aloud in the space of one minute. The test contains 168 non-vowelized words of an equivalent level of difficulty listed in columns. Words read correctly in the space of one minute are measured.
3	*Arithmetic Two-Minute test* - Participants’ mathematical automaticity skills were assessed using the Arithmetic Two-Minute test (Openhin-Bitton and Breznitz, unpublished). The task consists of 80 simple arithmetic calculation problems, including the four basic math operations (addition, subtraction, multiplication, and division). The problems are presented in four columns, 20 problems for each basic math operation. Participants are instructed to solve as many problems as possible, from all four types, in 2 min. Total time, accuracy and correct responses per minute are scored.
4	*DSM-5 attention/hyperactivity disorder questionnaire Hebrew version* (American Psychiatric Association, 2000) – was used to verify inclusion criteria of the ADHD group. The self-report questionnaire consists of 18 items 9 regarding inattentive symptoms and 9 regrading symptoms of impulsivity and hyperactivity. Each participant is asked to indicate for each item whether he or she experienced the particular symptom.

All participants underwent a series of cognitive tests to evaluate general intelligence as measured by Raven’s SPM tests^74^, reading^75^ and math skills. Details about these standardized tasks are presented in Table 1, and Results are shown in Table 2. The groups did not differ significantly in age, intelligence, and reading/math skills. Naturally, the ADHD group differed significantly from the control group in the ADHD measures derived from the DSM-5 questionnaire.

**Table 2:** Demographic and Psychometric Data of ADHD and Control Groups.

Measure	Group		*P*
	*ADHD* *Mean (SD)*	*Control* *Mean (SD)*	
Age (in years)	26.23 (3.03)	25.27 (2.46)	*0.12*
Raven’s SPM	50.64 (5.97)	52.45 (5.71)	*0.28*
Shatil reading test	94.56 (20.64)	103 (17.37)	*0.12*
Math skills	67.12 (8.13)	71.12 (10.77)	*0.14*
Inattentive symptoms	5.48 (2.5)	0.91 (1.9)	*0.00*
Hyperactive/impulsivity symptoms	5.20 (3.02)	1.37 (2.33)	*0.00*

### Apparatus and materials

Testing took place in a sound-attenuated chamber wherein participants were seated in front of a computer monitor during the entire experiment. Stimulus presentation and the recording of response time and accuracy were controlled by a computer program E-PRIME^76^. The stimulus material and card arrangements were similar to those used in the study of Foerde, *et al*.^50^ (see Fig. 5).

### Procedure

#### Probabilistic Learning Task

Participants carried out a probabilistic learning task, similar to that used in Foerde, *et al*.^50^. This task includes a training phase consisting of 100 trials, whereby on each trial, participants are presented with an Asian character (1 of 6 different characters) and are asked to predict which of two outcomes (different Hebrew letters) the Asian character is associated with (Fig. 5A). The probabilities used are such that each Asian character predicts that one of the two Hebrew letters yields a rewarding outcome on 83% of trials and the other letter on 17% of trials (Fig. 5C).

Following each response, feedback was presented after a fixed delay of either 0 s (*immediate feedback*), 3 s (*short delayed feedback*) or 6 s (*long delayed feedback*). The task was designed such that each Asian character was associated with one of the delay durations (two characters assigned randomly for each delay). Trial types for each feedback delay condition were interleaved throughout training. Participants were given up to 3 s to make a response. After responding, they were immediately shown their choice for 1 s, followed by the delay period (0, 3, or 6 s). The chosen outcome and character remained on screen during the delay to minimize working memory demands. Thus, the critical manipulation was the time interval between responses and feedback. Because response times could vary across trials and participants, the overall trial length (character onset to feedback end) could vary, but the time between responses and feedback stayed constant for each trial type. After the delay, performance feedback in the form of the words ‘correct’ or ‘incorrect’ was displayed for 1.5 s. The behavioral measurement of performance in the task entailed the percentage of successful choices for each feedback delay condition (i.e. selecting the letters that lead to correct feedback for each cue). Participants completed 180 learning trials of the task (learning phase), followed by a test phase, in which they were presented again with the previously presented Asian characters and were told to continue performing the task based on what they had learned. The testing phase was similar to the learning phase (180 trials), with the exception that no feedback was provided and that it started immediately following the learning phase, upon which instructions were presented on screen. (Fig. 5B).
